# ClickGene: an open cloud-based platform for big pan-cancer data genome-wide association study, visualization and exploration

**DOI:** 10.1186/s13040-019-0202-3

**Published:** 2019-06-26

**Authors:** Jia-Hao Bi, Yi-Fan Tong, Zhe-Wei Qiu, Xing-Feng Yang, John Minna, Adi F. Gazdar, Kai Song

**Affiliations:** 1 School of Chemical Engineering and Technology, Tianjin University, Tianjin, 300072 China; 2 School of Computer Software, Tianjin University, Tianjin, 300072 China; 3 Hamon Center for Therapeutic Oncology, University of Texas Southwestern Medical Center, Dallas, TX 75390 USA; 4 Department of Pharmacology, University of Texas Southwestern Medical Center, Dallas, TX 75390 USA; 5 Department of Internal Medicine, University of Texas Southwestern Medical Center, Dallas, TX 75390 USA; 6 Department of Pathology, University of Texas Southwestern Medical Center, Dallas, TX 75390 USA

## Abstract

Tremendous amount of whole-genome sequencing data have been provided by large consortium projects such as TCGA (The Cancer Genome Atlas), COSMIC and so on, which creates incredible opportunities for functional gene research and cancer associated mechanism uncovering. While the existing web servers are valuable and widely used, many whole genome analysis functions urgently needed by experimental biologists are still not adequately addressed. A cloud-based platform, named CG (ClickGene), therefore, was developed for DIY analyzing of user’s private in-house data or public genome data without any requirement of software installation or system configuration. CG platform provides key interactive and customized functions including Bee-swarm plot, linear regression analyses, Mountain plot, Directional Manhattan plot, Deflection plot and Volcano plot. Using these tools, global profiling or individual gene distributions for expression and copy number variation (CNV) analyses can be generated by only mouse button clicking. The easy accessibility of such comprehensive pan-cancer genome analysis greatly facilitates data mining in wide research areas, such as therapeutic discovery process. Therefore, it fills in the gaps between big cancer genomics data and the delivery of integrated knowledge to end-users, thus helping unleash the value of the current data resources. More importantly, unlike other R-based web platforms, Dubbo, a cloud distributed service governance framework for ‘big data’ stream global transferring, was used to develop CG platform. After being developed, CG is run on an independent cloud-server, which ensures its steady global accessibility. More than 2 years running history of CG proved that advanced plots for hundreds of whole-genome data can be created through it within seconds by end-users anytime and anywhere. CG is available at http://www.clickgenome.org/.

**Electronic supplementary material:**

The online version of this article (10.1186/s13040-019-0202-3) contains supplementary material, which is available to authorized users.

## Introduction

The rapid development of next-generation sequencing and array-based profiling methods now generate large quantities of diverse types of genomic data [1]. Correspondingly, public data portals like TCGA (The Cancer Genome Atlas) and COSMIC [2] provide more and more genome data in different formats and file types. While all of these enable researchers to study the genome at unprecedented resolution, due to the precondition of computer skills and mathematical/statistical techniques, analyzing of such big and diverse data sets is still a rate-limiting step in many studies. For instance, there are more than 32,555 cases in 310,859 files are available in GDC data portal. For a new user, it’s a very time-consuming process to figure out a way to download the proper data. Additionally, it is not reasonable for a given researcher to get familiar with all these kinds of trials of data downloading and analyzing. Therefore, a number of applications have been proposed to make it as friendly and timesaving as possible. For example: Tablet [3], BamView [4], IGV [5], MethylMix [6], GISTIC [7], Web-TCGA [8], TCGA-assembler [9], cBioPortal [10], GEPIA [11], The UCSC Cancer Genomics Browser [12] and so on.

Unfortunately, extremely varied requirements make it impossible for a given tool or platform to provide services to all kinds of end researchers. Consequently, even big consortium projects like TCGA and COSMIC provide only preprocessed data in different levels without any further analyzing; UCSC provides only different kinds of annotation information; TCGA-Assembler [9], GISTIC, MethylMix and GEPIA and so on provide only profiling or visualizing services for certain kinds of data type, e.g. MethylMix and GEPIA for only methylation and/or mRNA expression data; GISTIC for only CNV data.

Regarding to the profiling or visualizing web servers aiming for serving clinicians or experimental biologists, the number of them is increasing. But there are still many stumbling blocks. For instance, most of these applications require data downloading or package installation which makes them have requirements on users’ hardware and operation system configuration. Several available tools, such as cBioPortal [10], TCGA-Assembler [9] or Firebrowse (http://firebrowse.org/) require programming skills or show limitations when comparing multiple studies. Furthermore, none of the existing tools allow analyses based on the quantified difference of global CNV (copy number variation) patterns among pan-cancers since it has been proved that CNVs play important roles in histologic classifications of different cancer types [13–15]. Moreover, most of them were developed with R language instead of the professional web-developing languages, which limits their interaction and visualizing performance. More importantly, most of them are single-server based platform which makes their accessibility is not very stable nor accountable.

To overcome these bottlenecks, we developed the CG (ClickGene) platform (http://www.clickgenome.org/), a cloud-based one, to deliver fast and customizable functionalities to complement with the existing tools. Different from TCGA-Assembler which does not include any graphical interface, the graphical interface is an important integral part of CG. Several available DIY analysing tools make CG is different from Firebrowse since Firebrowse is only capable of displaying pre-calculated results. Indeed, Web-TCGA provides an infrastructure for comprehensive analyzing and visualizing the most common data types provided by TCGA. But besides the global profiling visualization for different data types, distributions of each individual gene are also available in CG. In addition, three new powerful plots (Mountain plot, Deflection plot and Directional Manhattan plot) and a similarity scoring method are proposed by us for analysing genome variations among different types of cancer. These methods are also available for analysing users own in-house data. More importantly, unlike other R-based web platforms, CG is developed with Java language. By making good use of ECharts, JavaScript and other professional web developing languages and MySQL database, several advanced plots can be created by only mouse-button clicking by end users. Additionally, Dubbo, a cloud distributed service governance framework for ‘big data’ stream global transferring, is used as the framework to develop CG platform. After being developed, CG is run on a cloud-server provided by an independent third-part cloud-service company, which ensures its accountably steady global accessibility. More than 2 years running history of CG proved that advanced plots for hundreds of whole-genome data can be created through it within seconds by end-users anytime and anywhere.

### Material and methods

#### The architecture of the cloud-based CG platform

**Fig. 1:**
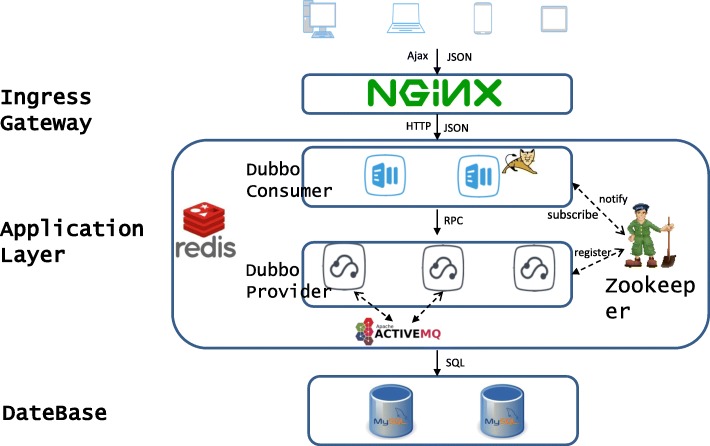
An overview of the CG cloud-based architecture

Bioinformatics research involves the storage of massive data, frequent calls, and compute-intensive algorithm analysis. Therefore, we adopted BootStrap (an open source toolkit for developing with HTML, and JavaScript. https://getbootstrap.com), jQuery (https://jquery.com), ECharts (https://github.com/apache/incubator-echarts) to build the front-end system for data parser and visualized interface with end-users. To make sure the high-performance, high-availability, high-concurrency are the features of the services provided by CG platform, the back-end of it was developed at the basis of a cloud-based distributed system architecture. In addition, our CG cloud-based platform combined Dubbo (http://dubbo.apache.org/en-us/), the integrative web application development SSM (SpringMVC, Spring, MyBatis) framework. An overview of the platform is presented in Fig. 1. Due to the limited space, only the brief introduction of each language and technique was given here. Details of them are available in the Additional file 1.

Dubbo is an RPC (Remote Procedure Call) service governance framework that enables our cloud-based platform to be distributed, modular and pluggable design and deployment. DispatcherServlet, as the core of the SpringMVC framework, is mainly used to intercept external requests and to distribute them to different controllers. According to the result processed by the controller, a corresponding response is generated and sent to the client. Spring is characterized by IOC (Inversion of Control) and AOP (Aspect Oriented Programming). Spring controls the life cycle of the object and the dependence between objects by IOC rather than directly by the program in the traditional implementation. AOP is implemented by a dynamic proxy. Java is a language for object-oriented programming. MyBatis is one of the most popular ORM (Object Relational Mapping) frameworks. The ORM automatically persists objects in a program to a relational database by means of metadata that describes the mapping between the object and the database. MySQL (https://www.mysql.com/), an open-source relational database management system (RDBMS), was used to manage all downloaded and preprocessed data. Additional file 2: Figure S1 shows a logical view of MySQL’s architecture. To make the database small enough, reduce disk I/O, and increase system throughput, we used table compression method. An example of the related data tables created in MySQL is shown in Additional file 2: Figure S2.

**Fig. 2:**
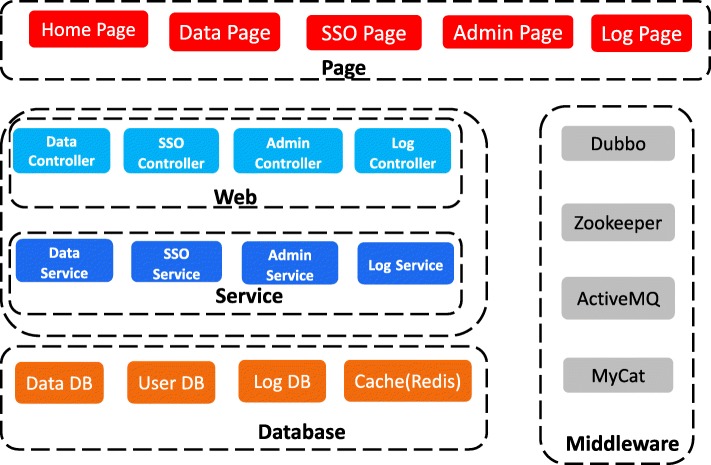
A profile of the functional module design of the entire system

Once a user inputs cancer or gene-related parameters at the front-end interface and submits a request, the back-end response processing is divided into three steps: receiving and allocating a request; processing a request, matching a method, statistically analyzing and processing the data; returning the result to the front-end. Finally, the front-end parses and visualizes the data. In short, user-server communication is implemented using TCP/IP sockets. A profile of the functional module design of the entire system is shown in Fig. 2.

Please see the Additional file 1 for more detailed introduction of these web-developing languages and toolkits. Detailed manual including in-house data uploading format instructions are available at the corresponding webpage at CG platform (http://www.clickgenome.org/guide/).

### Data processing

Over a decade after its initial funding, The Cancer Genome Atlas (TCGA) has generated vast amounts of data of 33 different kinds of primary tumor in different data types (including mRNA expression values, copy number variations, methylations and so on). Since it has included the most comprehensive information about the cancer landscape for researchers to further exploration, we downloaded and preinstalled TCGA public portal data in our database as examples to demonstrate the analyzing tools provided by CG platform. Up to now, all data was directly downloaded from the “Legacy GDC portal”. Considering about the different requirements on the data version, we provide two kinds of data updated before 10/05/2016 and 10/01/2018, respectively. It has been updated on a regular basis. Data analysis is restricted to autosomes. All non-primary samples are removed before any further pre-process and analysis. The available types of cancer in CG are listed in Additional file 3: Table S1. Since the TCGA (Legacy GDC) data format is still more familiar by end users, it was used as an example in this paper. The level 3 mRNA expression data in ‘*.rsem.genes.normalized_results’ files; the level3 CNV data in ‘*.hg19.seg.txt’ are used as examples in this paper. The average value of CNVs of all segments mapped to a specific gene partially or entirely is used as the CNV of it. Only HUGO symbols are acceptable for all CNV analyzing tools. Details of other provided TCGA and GDC data format are available in CG platform. Please go to Legacy GDC portal and GDC portal for more details of these data.

### Private in-house data

All analyzing tools provided by CG platform are available for analyzing users’ private in-house data. To secure the copyright of users uploaded private data, it will not be stored in our database for any further analysis. Detailed manual for data uploading format and examples are available at the corresponding webpage (http://www.clickgenome.org/guide/).

### Curve similarity analysis

According to the biological mechanism, CNVs of adjacent genes are closely related to each other. This means the position and ordering of CNV points of genes along chromosome arms can be seen as sequences or curves. To quantity the similarity of arm-wise to genome-wise CNV patterns between different types or trails of cancer, the curve similarity analysis was introduced as a measurement.

DTW (dynamic time warping) is a very widely used method for similarity analysis. In general, DTW is a sophisticated similarity measure that calculates an optimal match between two given sequences (e.g. time series) with certain restrictions. It can be non-trivially transformed. The sequences are “warped” non-linearly in time dimension to determine a measure of their similarity independent of certain non-linear variations. In genomic signals, after representing time instances by nucleotide positions and amplitude to the cumulated phase of signals, DTW is then suitable for adjustment of derived genomic signals [16–18]. For the same reason, it also can be used to measure the CNV curve similarity in Mountain plot.

DTW aligns sample values based on the minimization of the distance between pairs of samples. The values of accumulated distance are calculated from pairwise distances for each pair of samples in accordance with Eq.1.

1 $$ D\left(i,j\right)=\min \left[D\left(i-1,j-1\right),D\left(i-1\right)+d\left(i,j\right)\right] $$

where *D* symbolizes accumulated distance and *d* is a value of pairwise distance. The value of accumulated distance *D*(*i*, *j*) is determined by pairwise distance *d*(*i*, *j*) and minimum from the previous values of accumulated distances. This set of accumulated distances for each pair of samples forms a table which determines the criterion for alignment and repetition of samples. The result sequence warping is derived on the basis of minimization of the backward way from the right upper corner to the left lower corner.

DTW score is normalized to the range < 0, 1>. ‘0’ means the two sequences are completely different to each other while ‘1’ means they are basically coinciding with each other. Therefore, the closer the similarity to 1, the more similar they are to each other.

To test whether the inconsistent between the Mountain curves of two selected group samples are caused by random fluctuations, Bootstrapping test is provided to evaluate the significance of the differences between these two kinds of samples [19, 20]. Please see the Additional file 1 for more details.

Besides DTW, to quantity the similarity, we also introduced other three popular scores shown in the following equations:

2 $$ \mathrm{Distance}\ \mathrm{based}\ \mathrm{similarity}\ \mathrm{score}\ {\sum}_i\left({CNV}_{xi}-{CNV}_{yi}\right) $$

3 $$ \mathrm{Absolute}\ \mathrm{distance}\ \mathrm{based}\ \mathrm{similarity}\ \mathrm{score}\;{\sum}_i\left|{CNV}_{xi}-{CNV}_{yi}\right| $$

4 $$ \mathrm{Square}\ \mathrm{distance}\ \mathrm{based}\ \mathrm{similarity}\ \mathrm{score}\ {\sum}_i{\left({CNV}_{xi}-{CNV}_{yi}\right)}^2 $$

Please see the Additional file 1 or go to http://www.clickgenome.org for more details.

## Results

### The ClickGene (CG) user interface

There are six main web pages for users to explore CG platform and to use the provided functions:

***Home page****.* Displays an overview of this website, our team and all available analyses functions.

***Data Analysis****.* Provides all links to all provided tools using TCGA data as examples.

***Analyze yours****.* Users can analyze their own data with the tools provided by CG.

***User’s Guide****.* The detailed manual for users to apply the provided tools to analyze TCGA or their own in-house data. For user’s convenience, it is available in both HTML and PDF file types.

***Papers****.* A list of published research articles with the results obtained by using the analysis methods provided by CG.

***About us****.* Introduction of our lab, developing team and other members.

### The bioinformatics functions currently provided by CG

**Fig. 3:**
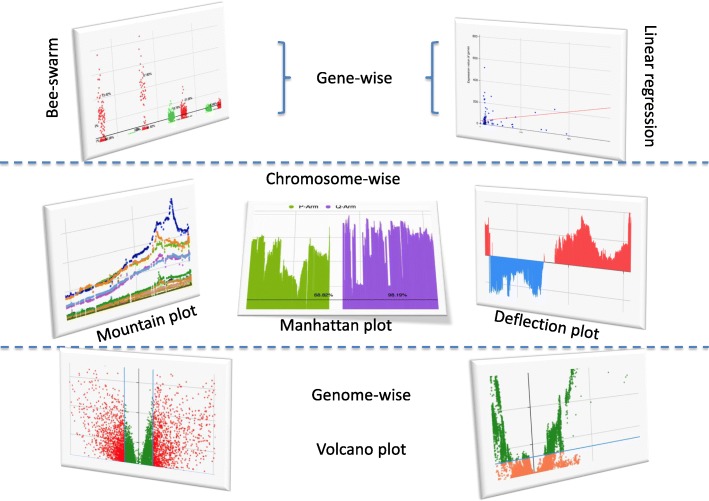
The example figures created by analyzing tools provided by CG

Making good use of the RPC (Remote Procedure Call) technique based on Dubbo for ‘big data’ stream service, the high speed big genome data global transferring was realized by CG platform. Along with the application of high sophisticated data pre-processing and data-mining methods, the following multiple level data profiling tools are provided by CG. The examples are shown in Fig. 3.

**Genome-wise profiling tool:** Volcano plot.

Volcano plot is a type of scatter-plot that is used to quickly identify changes in large data sets [21] [22]. It plots significance versus fold-change respectively on the *y* (−log10 of *p*-value) and *x* axes. The dashed horizontal line shows the cutoff of p-value (normally, *p* = 0.05) with points above it having *p* < cutoff and points below it having *p* > = cutoff.

### Chromosome-wise profiling tools

For illustrating chromosome or global expression/CNV patterns, CG provides different kinds of profiling and analyzing tools including: Mountain plot, Manhattan plot (directional/ regular) and ***Deflection plot***. The corresponding genome-wise profile can be obtained by combining these plots chromosome by chromosome. Please see the demonstration examples of them in the Discussion section. In these chromosome-wise plots, for chromosomes 13, 14, 15, 21, and 22, only genes on q arms are visible due to the fact that only them are represented on the microarray. Please see the Additional file 1 for more details.

***Mountain plo****t.* Mountain plot is named by Dr. Kai Song because its ups and downs look like mountain outlines seen from afar. It is a very useful scatter plot, invented by Dr. Adi F. Gazdar and Dr. Kai Song, originally for profiling genome-wide variations of copy numbers [13, 23]. In Mountain plot, each spot is the median/mean value of copy numbers of each gene in a group of cancer samples. The genes are sorted according to their genome locations. The space between two arms of each chromosome is the location of the corresponding centromere.

***Manhattan plot****.* Normally, in genome-wide association studies (GWAS), a Manhattan plot is a type of scatter plot, usually used to display data with a large number non-zero amplitude data-points [24]. To improve the feasibility and transmitting speed of our global website service, we did several modifications to the original Manhattan plot:

- Manhattan plot for genes rather than for SNPs (Single nucleotide polymorphisms), which means the Q-values are calculated for genes in two different groups of samples.
- Besides regular Manhattan plot, Directional Manhattan plot is provided as an option to show more information. If the median value of the gene in group1 is smaller than that in group2, the corresponding line is under the base line (normally, it’s zero line), otherwise, it is above the base line.

***Deflection plot****.* To Deflection plot, genomic coordinates are displayed along the *x-*axis. Each line stands for a gene. The amplitude of it stands for the negative logarithm of the Q-value, which is the significance testing value for each gene’s mRNA expression/copy number values in two concern groups (e.g. tumour samples in cancer type1 or cancer type2). Therefore, the more significant the difference between values in these two groups, the higher the y-axis value is. In addition, if the bigger variation for a gene is a negative one (median value in tumor samples is smaller than that in non-malignant samples), then the corresponding line is under the base line. Otherwise, it would be above the base line. Two default colors are assigned to these two types of cancer. Color1 indicates that the deflection (tumors vs. non-malignant samples) in cancer type1 is comparatively greater, whereas color2 indicates that the deflection in cancer type2 is comparatively greater. A gap within the individual chromosome data indicates the location of the centromere.

**Gene-wise visualization tools:** Bee-swarm plot (by gene/by cancer) and Linear regression analysis.

It’s very practical to have an overview of the distribution of mRNA expression values or CNVs of specific genes in a certain type of cancer or across different types of cancer. Therefore, Bee-swarm plot and linear regression analyses are provided. By making good use of these tools, CNV/expression profiles determined by tissue of origin of cancer and biomarker identification for different therapeutic purposes can be performed efficiently by experimental biologist or clinicians themselves.

The Bee-swarm plot is a one-dimensional scatter plot like “stripchart”, but with closely-packed, non-overlapping points. Here, the Bee-swarm plots with or without other options (e.g. box plot) are provided. In addition, the statistical hypothesis test (such as the student’s *t*-test) is also provided to test whether the difference in the values of the two concern groups is significant.

The Pearson correlation coefficient (PCC), the most widely used measure of the linear correlation between two variables ***X*** and ***Y***, is used to evaluate the linear relationship between mRNA expression values and CNVs of given genes.

## Discussion

The ever-increasing development of public cloud has revolutionized many traditional technology concepts and architectural design patterns. The use of the cloud can provide structural reliability, management convenience, cost controllability and service security.

Concurrent programming is a feature of the Java language. Concurrency refers to the ability to handle things in a unit of time. Especially for compute-intensive and IO-intensive programs, we can take full advantage of this feature to run multiple threads synchronously to complete computing tasks. Although concurrent programming has high requirements for developers, compared with R language, it can make full use of CPU resources, speed up program response, modularize development, and enable asynchronous calls. Therefore, we chose Java-based language to develop CG.

At present, front-end and back-end separation has become a standard way to develop Internet project. It can be realized by using Nginx (front-end server) and Tomcat (back-end server). The separation of front-end and back-end lays a solid foundation for large-scale distributed architecture, flexible computing architecture, multi-terminal services (browser, iPad, etc.) architecture and so on. In this architecture, the front-end and back-end projects are deployed independently. The front-end page invokes the back-end interface asynchronously and interacts with the back-end programs using JSON (JavaScript Object Notation, data format of the front-end interaction) data format. It speeds up the overall response. Even it causes more professional skills and a longer developing time, therefore, it was still chosen by our CG platform developing team for the better using experience of end-users and for the easier maintenance.

The cloud-based distributed system adopted by our back-end system has the following characteristics: multiple computers cooperate with each other and provide powerful service capability to the outside world; each internal computer can communicate with each other through the cloud; a request from a client to a server will go through multi-computers. Compared with the traditional monolithic architecture, consequently, it can increase the system capacity, enhance the system availability, make system modules more reusable, and make the system more scalable. The key of the distributed system is service governing and scheduling. Therefore, CG adopts Dubbo distributed service governance framework which can provide efficient service governance solutions.

At the initial stage of CG developing, SSM (SpringMVC, Spring and MyBatis) framework was just emerging and gradually gaining popularity, while SSH (Struts, Spring and Hibernate) was still the mainstream of Java web. The switch from SSH to SSM made us realize the vital importance of well-encapsulated generic modules. It proved the superiority of the front-end and back-end separation architecture.

Spring is a lightweight framework dedicated to various solutions for java web applications. Spring’s goals are: making existing technologies easy to use; promoting good programming habits. Spring is a comprehensive solution that adheres to the principle of ‘no building new wheels’. In the areas where there are already good solutions, Spring never repeats implementations but provides good support for them. Therefore, its openness lays a very good foundation for the updating of our CG platform.

The main existing technologies for operating databases are: JDBC (Java DataBase Connectivity), Hibernate and MyBatis. The main drawbacks of the traditional JDBC programming operation database technology are: the operation database has a big workload; the code coupling degree is high and the reusability is poor; the database connection resource needs to be manually closed which makes it a potential danger once it is forgotten to be closed. Hibernate and MyBatis are two major frameworks for the operational databases. They encapsulate the traditional JDBC operation database technology in different ways to overcome the above drawbacks of JDBC. Since Hibernate are unable to customize the assembly of SQL (Structured Query Language), weakly support for complex associations and complex SQL statements and hard to optimize SQL, it is not suitable for large-scale Internet high-performance requirements. Therefore, MyBatis was chosen as the framework for our operational database considering about its advantages such as: it is highly flexible; it can customize and optimize SQL based on the bottom layer; it has low learning threshold and is easy to maintain.

To meet the varied requirements of different potential users, Mountain plot, Manhattan plot and Deflection plot can be created using the median or mean values of a group of samples. In Manhattan plot and Deflection plot, Q-value instead of *p*-value is used to control the false discovery rate of the multiple hypothesis testing [25]. Additionally, user’s can control it by specifying a different significance level rather than the defaulted 0.05. For example, according to the Bonferroni correction (the most conservative family-wise error control), the significance level is 0.05/*n*, (*n* is the number of independent hypothesis testing) [26, 27]. Even though, Volcano plot is a multiple hypothesis testing too, it’s still the p-value rather an Q-value is more widely used in this plot.

To verify the performance of CG, we firstly applied the provided tools to a collection of TCGA GBM (Glioblastoma multiforme) samples due to the fact that those functional roles of a substantial number of copy number alterations have already been validated in preclinical models [13, 28, 29].

**Fig. 4:**
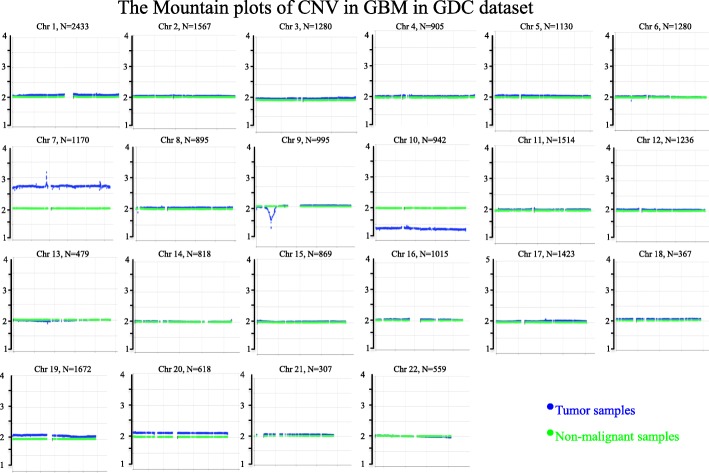
The genome-wise Mountain plots of CNV in GBM in GDC dataset

Since MEAN value is particularly sensitive to outliers, we used median-value-Mountain-plot as an example to illustrate the process of using tools provided by CG. Figure 4 shows the CNV Mountain plots of the first 22 chromosomes of GBM in TCGA. Table 1 lists the DTW scores and the corresponding Bootstrapping test *p*-values of each arm of the entire genome of it.

**Table 1:** The DTW scores and the corresponding Bootstrapping test values for GBM

Chr	Arm	GBM		Chr	Arm	GBM	
		DTW	*p*-value			DTW	p-value
1	P-Arm	0.98	< 0.1E-05	11	P-Arm	0.99	< 0.1E-05
1	Q-Arm	0.97	< 0.1E-05	11	Q-Arm	0.99	< 0.1E-05
2	P-Arm	0.98	< 0.1E-05	12	P-Arm	0.97	< 0.1E-05
2	Q-Arm	0.98	< 0.1E-05	12	Q-Arm	0.99	< 0.1E-05
3	P-Arm	0.98	< 0.1E-05	13	Q-Arm	0.99	< 0.1E-05
3	Q-Arm	0.96	< 0.1E-05	14	Q-Arm	1.00	8.00E-03
4	P-Arm	0.98	< 0.1E-05	15	Q-Arm	0.99	3.00E-04
4	Q-Arm	0.98	< 0.1E-05	16	P-Arm	0.99	< 0.1E-05
5	P-Arm	0.97	< 0.1E-05	16	Q-Arm	0.99	< 0.1E-05
5	Q-Arm	0.97	< 0.1E-05	17	P-Arm	0.98	< 0.1E-05
6	P-Arm	0.99	< 0.1E-05	17	Q-Arm	0.98	< 0.1E-05
6	Q-Arm	0.99	< 0.1E-05	18	P-Arm	0.97	< 0.1E-05
7	P-Arm	0.67	< 0.1E-05	18	Q-Arm	0.97	< 0.1E-05
7	Q-Arm	0.67	< 0.1E-05	19	P-Arm	0.96	< 0.1E-05
8	P-Arm	0.99	< 0.1E-05	19	Q-Arm	0.95	< 0.1E-05
8	Q-Arm	0.97	< 0.1E-05	20	P-Arm	0.92	< 0.1E-05
9	P-Arm	0.88	< 0.1E-05	20	Q-Arm	0.92	< 0.1E-05
9	Q-Arm	0.98	< 0.1E-05	21	P-Arm	1.00	1.68E-02
10	P-Arm	0.80	< 0.1E-05	21	Q-Arm	0.97	< 0.1E-05
10	Q-Arm	0.69	< 0.1E-05	22	Q-Arm	1.00	7.00E-04

From Fig. 4, it is very clear to see the amplifications and deletions. There are three broad amplifications: 7, 19 and 20. Among them, 7, 19p and 20 are proved by other publications [30–32]. The broad amplification of Chr 7 with overlapping focal EGFR (7p11.2) amplification is a very well known one (shown in Additional file 2: Figure S3) [7, 30].

According to the Beeswarm plots shown in Additional file 2: Figure S4, we could see that the copy numbers of EGFR in 43.7% (including one outlier) of GEM tumour smaples are higher than 4. These results strongly confirm the focal gains on this gene. The patient ID of outlier of EGFR is TCGA-06-0187-01 and the corresponding copy number is 151.4319. Its expression value is 86,384.59 which is the second highest one in the Beeswarm plot of this gene in GBM tumour samples. These results indicate that this patient needs a further checking.

Besides these broad amplifications, there are broad deletions on 9p and 10, which are very obvious and consistent with what have been reported by other papers [30]. The broad deletion of 9p with overlapping focal CDKN2A/B (9p21.3) deletion is also a very well-known one [13]. Additional file 2: Figure S5 show that: ESCA, HNSC and LUSC in SCCs; PAAD in ADCs; BLCA in other epithelial; GBM, MESO and THYM in non-epithelial cancer types all have CDKN2A/B focal deletion. Among them, GBM shows the greatest deletion, then the MESO. The relationship among these primary tumors may be worthy of further research.

Compared with MEAN value, MEDIAN value is more robust. This is the reason that fewer amplifications and deletions were found by using median-value-Mountain-plot.

In our previous study about Genome-wide copy number variation patterns, we analysed the differences among CNV patterns in LUAD, LUSC and other histologically similar tumours arising at other sites. But all these analyses are qualitative rather than quantitative. Therefore, we introduced curve similarity analysis to quantity the variants among different cancer types. Together with Bootstrapping test, both the similarity and the significance of the Mountain curves can be quantified.

Among these methods, DTW is a normalized measurement for the scores calculated with it are scaled to [0, 1]. Due to the limited space, we used only DTW scores as examples to measure the differences in CNV patterns among different cancer types.

According to the definition of DTW score, we could see that the length of the alteration is more important than the height of it. Taking GBM as an example: there are about 400 more genes located in 7q than in 7p. Even with one big focal amplicon on 7p, its DTW score is 0.67 (*p* < 0.1E-05 for Bootstrapping test) while the DTW score for the longer 7q is also 0.67 (p < 0.1E-05 for Bootstrapping test). Therefore, it’s better to use it together with Mountain plot, Deflection plot and Manhattan plot for more details.

Additional file 4: Table S2 shows the genome-wide arm-wise DTW scores and the corresponding *p*-values of main ADCs and SCCs. For each type of cancer, the DTW score is the difference between the tumor group and the corresponding non-malignant samples. If we take ADCs as one kind and SCCs as another kind, the biggest difference between CNV patterns of these two kinds happens on 3q. Additional file 2: Figure S6 shows their Mountain plots of 3q. All SCCs have big arm-wise amplifications on 3q while ADCs (except for OV) have only very mild ones.

From Additional file 4: Table S2, we could see that OV has the smallest genome-wide DTW score (19.63) among ADCs and SCCs. Bootstrapping test shows that the CNV pattern across the whole genome is also significantly different from that of non-malignant group. This result indicated that among all these primary tumors, CNV pattern of OV is the most varied one compared with non-malignant samples. LUSC (DTW = 20.44) and READ (DTW = 20.45) are the second and third varied one, respectively.

The Mountain plots of ADCs and SCCs on 5p, 20q and Chr13 are shown in Additional file 2: Figures S7, S8 and S9, respectively. From Additional file 2: Figure S7 we could see that genes located on 5p in LUSC, CESC, CHOL, LUAD and OV show arm-wise amplifications. Among them, CNV patterns have the highest median values in LUSC tumor samples. The Mountain plot on 20q in OV, COAD and READ shown in Additional file 2: Figure S8 show arm-wise amplifications. Among them, CNV patterns have the highest mean values in READ tumor samples. It means copy number pattern of 20q in READ tumor is most different from non-malignant samples [33, 34]. Additional file 2: Figure S9 shows great amplification on Chr13 in READ and COAD tumor samples while those of other ADCs and SCCs are very similar to those of the control samples. Additionally, OV shows arm-wise deletion on 13p. These figures strongly confirmed that DTW score could quantify the CNV similarity correctly.

The DTW scores of THCA across the entire genome are almost equal to 1 but some of p-vaules are smaller than 0.05, which means that it almost has no copy number alterations (the distributions may have different standard deviation values). This confirms the results found in ref. [35]. Only Chr 22 of it is a little bit different from the non-malignant samples whose DTW score is 0.99 (shown in Additional file 2: Figure S10).

## Conclusions

The cloud-based distributed service governance framework for ‘big data’ stream global transferring and calculating make it possible to store and transfer data as big as genome SNPs and so on in several seconds. Using high sophisticated Java, ECharts and other web developing and database management techniques, together with advanced data mining technology, the developed cloud-based CG platform enables end-users without any computational programming skills to perform a diverse range of gene expression and CNV analyses. Three new methods created by us and the introduced similarity scores are very helpful for specific profiling or gene targeting clinical analysis. The performance of it is strongly confirmed by the results consistent with what has been proved by published research. By using it, therefore, end-users can easily explore the large TCGA data and their own private in-house datasets for any specific pan-cancer analysis. Meanwhile, the customizable parameters of CG also enable users to extensively customize the results and the visualization. It complements well with other available tools such as MethylMix, GEPIA and so on. With the continuous further enhancement, CG platform has the potential to become an integral part of routine data analyses for experimental biologists and clinicians.

## Additional files

Additional file 1: Details of languaging tools, methods and examples. (DOCX 55 kb)

Additional file 2: **Figure S1.** A logical view of MySQL server architecture. **Figure S2.** The ERS (Entity-relationship model) for describing the relationship between data tables saved in MySQL database. **Figure S3.** A) Mountain plot of Chr7 of GBM in GDC dataset. **Figure S4.** The Beeswarm plots of copy numbers of gene EGFR in GBM tumor and non-malignant samples. **Figure S5.** Mountain plot 9p with CDKN2A/B focal deletions. **Figure S6.** Mountain plots of Chr3 in ADCS and SCCS. **Figure S7.** Mountain plot of 5p of all available ADCS and SCCS in GDC dataset. **Figure S8.** Mountain plot of Chr20 of all available ADCs and SCCs in GDC dataset. **Figure S9.** Mountain plot of Chr13 of all available ADCs and SCCs in GDC dataset. **Figure S10.** Mountain plot of Chr22 of THCA in GDC dataset. **Figure S11.** Volcano plots of copy numbers and mRNA expression values in LUAD vs LUSC. *y* axis is *p*-value of significance test (usually base 10). The *x* axis is the log of the fold change between the two conditions. (PPTX 2610 kb)

Additional file 3: **Table S1.** The available types of cancer in CG are listed. (XLSX 12 kb)

Additional file 4: **Table S2.** The arm-wise DTW scores for ADCs and SCCs. (XLSX 38 kb)

## Data Availability

The datasets generated and/or analysed during the current study are available in the Legacy GDC (https://portal.gdc.cancer.gov/legacy-archive/search/f) and ClickGene (http://www.clickgenome.org/welcome/) repositories.

## Abbreviations

ACC: Adrenocortical Carcinoma
ADC: Adenocarcinoma
BLCA: Bladder Urothelial Carcinoma
BRCA: Breast Carcinoma
CESC: Cervical Squamous Cell Carcinoma and Endocervical Adenocarcinoma
CHOL: Cholangiocarcinoma
CNV: Copy Number Variation
COAD: Colon Adenocarcinoma
CRCA: Colorectal Carcinoma
DLBC: Lymphoid Neoplasm Diffuse Large B-Cell Lymphoma
DSF: Distributed Services Framework
DTW: Dynamic Time Warping
ESCA: Esophageal Carcinoma
ESSC: Esophageal Squamous Cell Carcinoma
GBM: Glioblastoma
GDC: Genomic Data Commons Data Portal
GWAS: Genome-Wide Association Study
HNSC: Head and Neck Squamous Cell Carcinoma
KICH: Kidney Chromophobe
KIRC: Kidney Renal Clear Cell Carcinoma
KIRP: Kidney Renal Papillary Cell Carcinoma
LAML: Acute Myeloid Leukemia
LCC: Large Cell Cancer
LGG: Brain Lower Grade Glioma
LIHC: Liver Hepatocellular Carcinoma
LOH: Loss of Heterozygosity
LUAD: Lung Adenocarcinoma
LUSC: Lung Squamous Cell Carcinoma
MESO: Mesothelioma
NGS: Next-Generation Sequencing
NSCLC: Non-Small Cell Lung Cancer
OV: Ovarian Serous Cystadenocarcinoma
PAAD: Pancreatic Adenocarcinoma
PCPG: Pheochromocytoma and Paraganglioma
PRAD: Prostate Adenocarcinoma
READ: Rectum Adenocarcinoma
RPC: Remote Procedure Call
SARC: Sarcoma
SCC: Squamous Cell Carcinoma
SKCM: Skin Cutaneous Melanoma
SNP: Single nucleotide polymorphism
STAD: Stomach Adenocarcinoma
TCGA: The Cancer Genome Atlas
TGCT: Testicular Germ Cell Tumors
THCA: Thyroid Carcinoma
THYM: Thymoma
UCEC: Uterine Corpus Endometrial Carcinoma
UCS: Uterine Carcinosarcoma
