# Supplementary material for: ClickGene: an open cloud-based platform for big pan-cancer data genome-wide association study, visualization and exploration
Source: BioData Min. 2019 Jun 26;12:12. doi: 10.1186/s13040-019-0202-3 (PMC6595587; doi:10.1186/s13040-019-0202-3)
Supplement: Supplementary file 1 — Details of languaging tools, methods and examples. (DOCX 55 kb) [file 13040_2019_202_MOESM1_ESM.docx]

**Supplementary Document**

**ClickGene: an open cloud-based platform for big pan-cancer data genome-wide association study, visualization and exploration**

Jia-Hao Bi^1#^, Yi-Fan Tong^1#^, Zhe-Wei Qiu^1^, Xing-Feng Yang^2^, John Minna^3,4,5^, Adi F. Gazdar^3,6^, Kai Song^1,3＊^

^1^ School of Chemical Engineering and Technology, Tianjin University, Tianjin, 300072,China

^2^ School of Computer Software, Tianjin University, Tianjin, 300072, China

^3^Hamon Center for Therapeutic Oncology, University of Texas Southwestern Medical Center, 75390, Dallas, Texas, USA

^4^Department of Pharmacology, University of Texas Southwestern Medical Center, 75390, Dallas, Texas, USA

^5^Department of Internal Medicine, University of Texas Southwestern Medical Center, 75390, Dallas, Texas, USA

^6^Department of Pathology, University of Texas Southwestern Medical Center, 75390, Dallas, Texas, USA

* To whom correspondence should be addressed. Tel: +86-22-27403389; Fax: +86-22-27403389; Email: [ksong@tju.edu.cn](mailto:ksong@tju.edu.cn)

**Brief introduction of these web-developing languages and toolkits**

*HTML.* (HyperText Markup Language), a standard markup language for creating web pages.

*CSS.* (Cascading Style Sheets)[1], a computer language used to add style (fonts, spacing, color, etc.) to structured documents (such as HTML documents or XML applications).

*JavaScript.* [2], a high-level, [interpreted](https://www.wikiwand.com/en/Interpreted_language) [programming language](https://www.wikiwand.com/en/Programming_language).

*ECharts.* [3], a free, powerful charting and visualization library offering an easy way of adding intuitive, interactive, and highly customizable charts [3].

*jQuery.* ([https://jquery.com](https://jquery.com/)), a fast, small, and feature-rich JavaScript library.

*Bootstrap* ([https://getbootstrap.com](https://getbootstrap.com/)), a most popular HTML, CSS, and JavaScript framework for developing responsive, mobile-first websites.

*Ajax.* [3] (Asynchronous Javascript And XML), the official name is XMLHttpRequest,  a set of web development techniques using many Web technologies on the client side to create asynchronous web applications.

*Nginx.* (<https://www.nginx.com/resources/wiki/>), Nginx is a high-performance HTTP server written by Russians. It plays three roles: the front-end *HTTP.* (Hypertext Transfer Protocol) accessor; a load balancer with simple configuration; a solver to JavaScript cross-domain issues in a front-end and back-end separation architecture through reverse proxy configuration.

*Dubbo.* (<http://dubbo.apache.org/>), a high-performance, java based RPC (Remote Procedure Call) framework open-sourced by Alibaba.

*Dubbo Consumer*. A service consumer is responsible for invoking the service.

*Dubbo Provider*. A service provider implements the services defined in the interface, that is, the implementation of the service.

*Registry*. Zookeeper(<http://zookeeper.apache.org/>) plays a role as a registry which is used for automatic service registration and discovery.

*RDB.* (Relational Database)[4], a digital [database](https://www.wikiwand.com/en/Database) based on the [relational model](https://www.wikiwand.com/en/Relational_model) of data.

*MySQL*. (<https://www.mysql.com/>), an open-source relational database management system (RDBMS), was used to manage all downloaded and processed data.

**A profile of the functional module design of the entire system.**

The functions of the whole system can be roughly divided into five parts:

1) Page: The front-end pages can be divided into five functional modules.

Home Page: Main page of CG cloud-based platform.

Data Page: Interact with users to analyze and visualize data, including two interface web-pages: ‘Data Analysis’ and ‘Analyze Yours’.

SSO Page: User registration page.

Admin Page: A management page for the website to maintain the personnel rights' control.

Log Page: Track user’s moves and monitor user’s traffic.

2) Web: This part (four modules) receives the request and. match the corresponding methods that call the next part according to the request address.

Data Controller: Receive data analysis related operations.

SSO（Single Sign-on）Controller: Receive user’s registration, send an activating email and other related operations.

Admin Controller: Receive website maintenance personnel permission control related operations.

Log Controller: Receive operations that monitoring user’s visits and tracking user’s moves.

3). Service: This part (four modules) is a series of business-related operations including the operations on the Database part and method calls used by the Web part.

Data Service: Process data analysis related operations.

SSO Service: Handle user’s registration and email activation related operations.

Admin Service: Deal with the actions that related to the control of website maintenance personnel rights.

Log Service: Process operations that monitor user’s traffic and track user’s actions.

4).Database：This part (four modules) is for managing and storing data.

User DB (DataBase): Store user related information and website maintenance personnel authority control related information.

Data DB: Store bioinformatics-related data.

Log DB: Store logs and user action-related data.

Redis: an in-memory database used to cache data to improve website access performance.

5).Middleware (Middleware.org): A computer software that provides services to software applications beyond those available from the operating system.

Dubbo: An RPC framework to provide RPC communication.

Zookeeper: An open source distributed coordination service that acts as a registry.

ActiveMQ: is the most popular and powerful open source messaging and [Integration Patterns](http://activemq.apache.org/enterprise-integration-patterns.html) server.

**Linear regression analysis**

In statistics, **linear regression** is a linear approach for modelling the relationship between a scalar dependent variable *y* and an independent variables *x*. The case of one explanatory variable is called simple linear regression. It can be applied to quantify the strength of the relationship between *y* and the *x* to assess whether *x* may have no relationship with *y* at all or contain redundant information about *y*.

It is very useful to see whether copy number variations and the corresponding mRNA expression values for a certain gene are linearly related to each other. Therefore, a linear regression analysis function is provided by CG platform. To do this, the **Pearson correlation coefficient (PCC)** is calculated as a measurement.

The **Pearson correlation coefficient (PCC)**, also referred to as**Pearson's *r***, is a most widely used measure of the linear correlation between two variables ***X*** and ***Y***. It has a value between +1 and −1, where 1 is total positive linear correlation, 0 is no linear correlation, and −1 is total negative linear correlation [5, 6].

Pearson's correlation coefficient when applied to a sample is commonly represented by the letter r and may be referred to as the sample correlation coefficient or the sample Pearson correlation coefficient. We can obtain a formula for *r* by substituting estimates of the covariances and variances based on a sample into the formula above. So if we have one dataset of copy numbers $\left\{ x_{1},\cdots,x_{n} \right\}$ containing n values and another dataset of the corresponding mRNA expression values $\left\{ y_{1},\cdots,y_{n} \right\}$ containing n values then that formula for *r* is:

$r=\frac{\sum_{i=1}^{n} (x_{i}-\bar{x})(y_{i}-\bar{y})}{\sqrt{\sum_{i=1}^{n} {(x_{i}-\bar{x})}^{2}}\sqrt{\sum_{i=1}^{n} {(y_{i}-\bar{y})}^{2}}}$ (1)

where n is the sample size, $x_{i}$ and $y_{i}$ are the single samples indexed with $i$, $\bar{x}=\frac{1}{n}\sum_{i=1}^{n} x_{i}$ (the sample mean) and analogously for $\bar{y}$.

**Bootstrapping based significance test for DTW**

To test whether the inconsistent between the Mountain curves of two different groups of samples are caused by random fluctuations, Bootstrapping based significance test is provided to evaluate the significance of the difference between these two kinds of samples [7, 8].

For CNV genome datasets of a group with *m* samples and of another group with *n* samples (the DTW score between them is noted as DTW*_tn_*), the steps for Bootstrapping test are as follows:

1. Mix all samples into one group;
2. Randomly rearrange the samples of the mixed group;
3. Select the first *m* samples as group1 and the rest as group2;
4. Calculate the DTW score between group1 and group2;
5. Repeat step 2-5 10,000 times and get the DTW_1…10000_ scores
6. Sort DTW*_tn_* and all 10000 DTW scores together, if DTW*_tn_* ranked as the *i*th, then the corresponding p-value is *i*/10000.

If p-value<0.05, the DTW score between these two groups of samples are not considered to be caused by random fluctuations.

**Datasets**

For mRNA expression status, RNA-SeqV2 (RNA-Seq by Expectation Maximization, RSEM) data was measured by the Illumina HiSeq 2000 RNA Sequencing Version 2 platform. It is suggested to provide more accurate results [9] for down-stream analysis for taking transcript length into account. Therefore, the level 3 mRNA expression data in ‘*.rsem.genes.normalized_results’ files was used in CG without any further processing. In these TCGA format files, gene IDs are in the format of Entrez/LocusLink gene symbols followed by Entrez/LocusLink gene IDs, *e.g*. ‘EGFR|1956’. Since Entrez symbols and HUGO (Human Genome Organization) symbols used in UCSC annotation system are both widely used, they are both acceptable for the mRNA expression analysing tools provided with CG platform. Information provided by Genecards (<https://www.genecards.org/Guide/Search)> is used as the reference whenever there is an inconsistence between these two gene symbol systems. Because the level 3 mRNA expression data of TCGA was log2 transformed in default, CG platform provide options for users to choose whether to use the log2 transformation for comparison or not. For Legacy TCGA level 3 CNV data measured by Affymetrix Genome-Wide Human SNP Array 6.0, two kinds of CNV data are provided in ‘*.hg19.seg.txt’ and ‘*.hg19.nocnv.seg.txt’ files. The CNV data in 'nocnv' files indicates that a fixed set of probes that frequently contain germ line CNVs are removed prior to segmentation. Since CNV data in ‘*.hg19.seg.txt’ contains all measurable segments information, therefore, it was used in CG and mapped to the annotation file downloaded from UCSC (http://hgdownload.cse.ucsc.edu/goldenPath/hg19/database/refGene.txt.gz) to get the CNV for protein coding genes. The average value of CNVs of all segments mapped to a specific gene partially or entirely was used as the CNV of it. Only HUGO symbols are acceptable for all CNV analysing tools.

**Application in pan cancer copy number pattern analysis**

Lots of efforts have been made to distinguish LUAD from LUSC to improve the outcomes of diagnosing and treating of NSCLC. In our previous study, we created Matlab version of Mountain plot and Deflection plot to do this analysis too [10]. Considering about the visualizing performance of these two kinds of plots, the cloud-based version of them are available in ClickGene. Due to the limited space, they are not shown here. The Volcano plots of mRNA expression values and copy numbers of them are shown in Figure S11. From it, we could see that: most genes have significantly different CNV and mRNA expression distributions in LUAD and LUSC. But unlike the mRNA expression values, the amplitude changes in CNV patterns between these two different subtypes are hardly significant.

**List of abbreviations**

ACC Adrenocortical Carcinoma

ADC Adenocarcinoma

BLCA Bladder Urothelial Carcinoma

BRCA Breast Carcinoma

CESC Cervical Squamous Cell Carcinoma and Endocervical Adenocarcinoma

CHOL Cholangiocarcinoma

CNV Copy Number Variation

COAD Colon Adenocarcinoma

CRCA Colorectal Carcinoma

DLBC Lymphoid Neoplasm Diffuse Large B-Cell Lymphoma

DTW Dynamic Time Warping

DSF Distributed Services Framework

ESCA Esophageal Carcinoma

ESSC Esophageal Squamous Cell Carcinoma

GBM Glioblastoma

GDC Genomic Data Commons Data Portal

GWAS Genome-Wide Association Study

HNSC Head and Neck Squamous Cell Carcinoma

KICH Kidney Chromophobe

KIRC Kidney Renal Clear Cell Carcinoma

KIRP Kidney Renal Papillary Cell Carcinoma

LAML Acute Myeloid Leukemia

LCC Large Cell Cancer

LGG Brain Lower Grade Glioma

LIHC Liver Hepatocellular Carcinoma

LOH Loss of Heterozygosity

LUAD Lung Adenocarcinoma

LUSC Lung Squamous Cell Carcinoma

MESO Mesothelioma

NGS Next-Generation Sequencing

NSCLC Non-Small Cell Lung Cancer

OV Ovarian Serous Cystadenocarcinoma

PAAD Pancreatic Adenocarcinoma

PCPG Pheochromocytoma and Paraganglioma

PRAD Prostate Adenocarcinoma

READ Rectum Adenocarcinoma

RPC Remote Procedure Call

SARC Sarcoma

SCC Squamous Cell Carcinoma

SNP Single nucleotide polymorphism

SKCM Skin Cutaneous Melanoma

STAD Stomach Adenocarcinoma

TCGA The Cancer Genome Atlas

TGCT Testicular Germ Cell Tumors

THCA Thyroid Carcinoma

THYM Thymoma

UCS Uterine Carcinosarcoma

UCEC Uterine Corpus Endometrial Carcinoma

1. Meyer E: *Cascading Style Sheets: The Definitive Guide.* O'Reilly Media; 2000.

2. Flanagan D: *JavaScript: The Definitive Guide.* O'Reilly Media, Inc; 2006.

3. Li D, Mei H, Shen Y, Su S, Zhang W, Wang J, Zu M, Chen W: **ECharts: A declarative framework for rapid construction of web-based visualization.** *Visual Informatics*.

4. Codd EF: **A relational model of data for large shared data banks.** *Commun ACM* 1970, **13:**377-387.

5. Wang YP, Li KB: **Correlation of expression profiles between microRNAs and mRNA targets using NCI-60 data.** *BMC Genomics* 2009, **10:**218.

6. Patnaik SK, Dahlgaard J, Mazin W, Kannisto E, Jensen T, Knudsen S, Yendamuri S: **Expression of microRNAs in the NCI-60 cancer cell-lines.** *PLoS One* 2012, **7:**e49918.

7. Jaki T, Su TL, Kim M, Lee Van Horn M: **An evaluation of the bootstrap for model validation in mixture models.** *Commun Stat Simul Comput* 2018, **47:**1028-1038.

8. Efron B: **Bootstrap Methods: Another Look at the Jackknife.** *The Annals of Statistics* 1979, **7:**1-26.

9. Li B, Dewey CN: **RSEM: accurate transcript quantification from RNA-Seq data with or without a reference genome.** *BMC Bioinformatics* 2011, **12:**323.

10. Qiu ZW, Bi JH, Gazdar AF, Song K: **Genome-wide copy number variation pattern analysis and a classification signature for non-small cell lung cancer.** *Genes Chromosomes Cancer* 2017, **56:**559-569.
