# Supplementary material for: ClickGene: an open cloud-based platform for big pan-cancer data genome-wide association study, visualization and exploration
Source: BioData Min. 2019 Jun 26;12:12. doi: 10.1186/s13040-019-0202-3 (PMC6595587; doi:10.1186/s13040-019-0202-3)
Supplement: Supplementary file 2 — Figure S1. A logical view of MySQL server architecture. Figure S2. The ERS (Entity-relationship model) for describing the relationship between data tables saved in MySQL database. Figure S3. A) Mountain plot of Chr7 of GBM in GDC dataset. Figure S4. The Beeswarm plots of copy numbers of gene EGFR in GBM tumor and non-malignant samples. Figure S5. Mountain plot 9p with CDKN2A/B focal deletions. Figure S6. Mountain plots of Chr3 in ADCS and SCCS. Figure S7. Mountain plot of 5p of all available ADCS and SCCS in GDC dataset. Figure S8. Mountain plot of Chr20 of all available ADCs and SCCs in GDC dataset. Figure S9. Mountain plot of Chr13 of all available ADCs and SCCs in GDC dataset. Figure S10. Mountain plot of Chr22 of THCA in GDC dataset. Figure S11. Volcano plots of copy numbers and mRNA expression values in LUAD vs LUSC. y axis is p-value of significance test (usually base 10). The x axis is the log of the fold change between the two conditions. (PPTX 2610 kb) [file 13040_2019_202_MOESM2_ESM.pptx]

## Slide 1
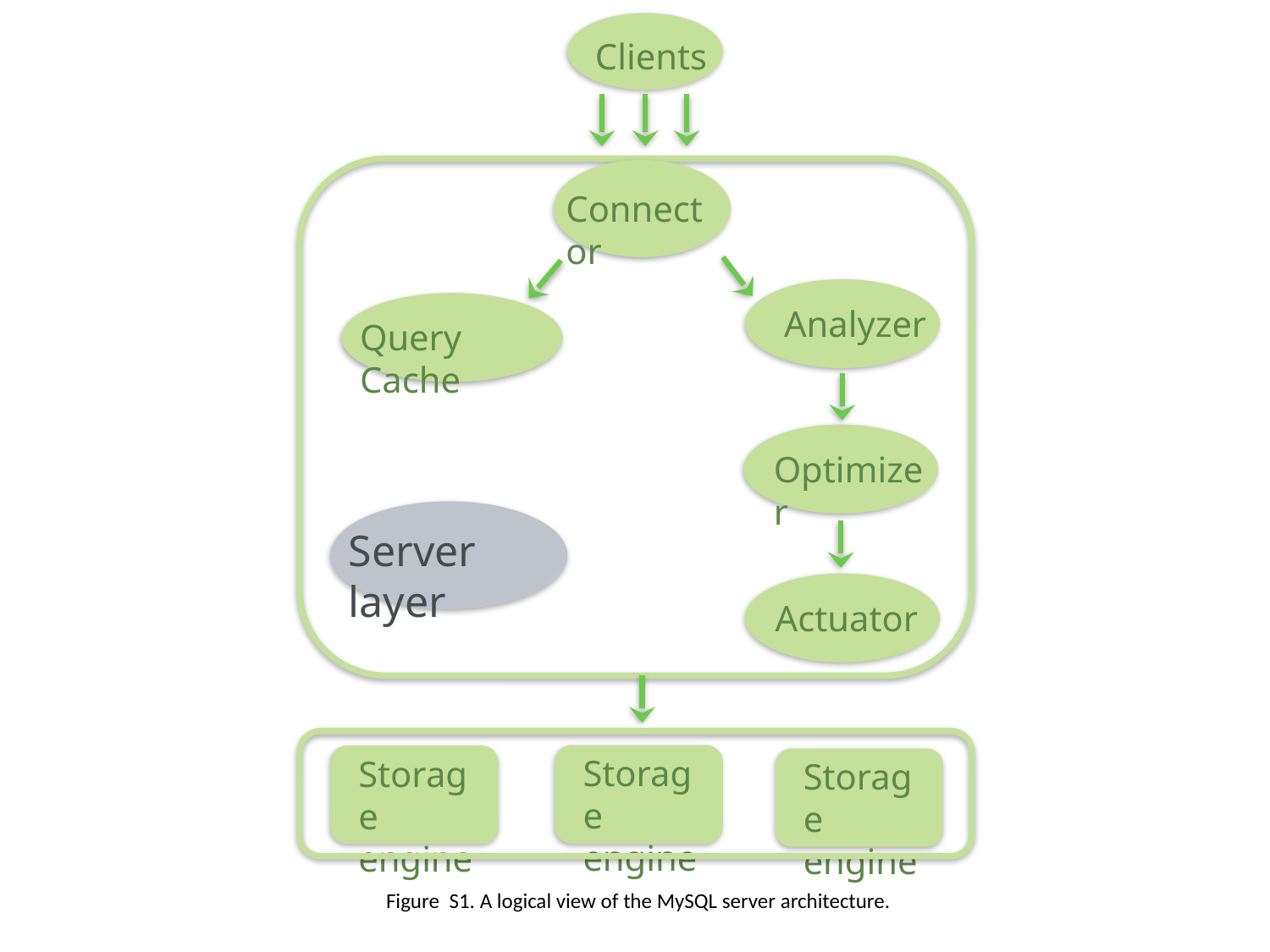

Clients
Connector
Analyzer
Query Cache
Optimizer
Server layer
Actuator
Storage
engine
Storage
engine
Storage
engine
Figure S1. A logical view of the MySQL server architecture.

## Slide 2
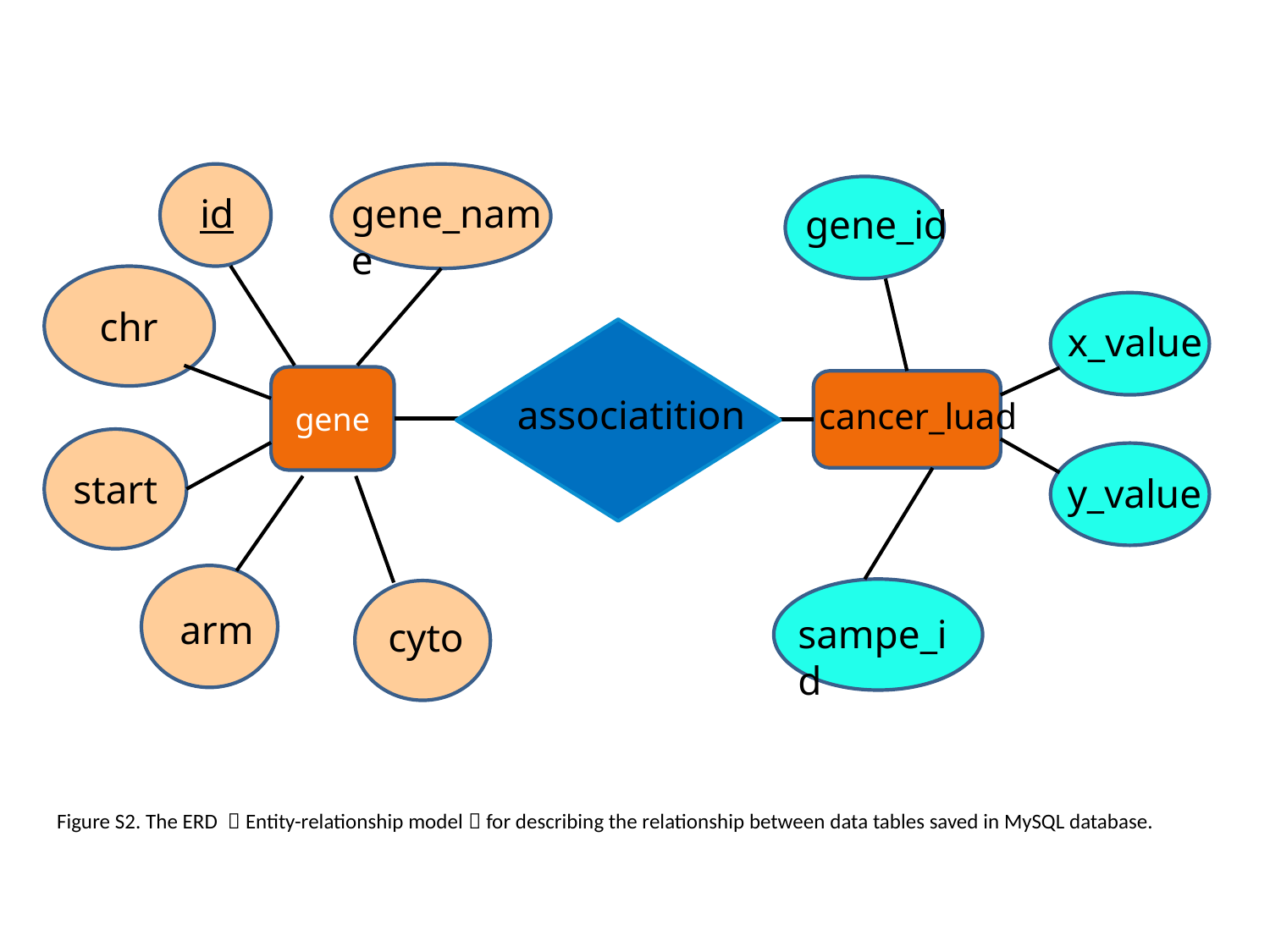

gene_name
id
gene_id
chr
x_value
gene
associatition
cancer_luad
start
y_value
arm
sampe_id
cyto
Figure S2. The ERD （Entity-relationship model）for describing the relationship between data tables saved in MySQL database.

## Slide 3
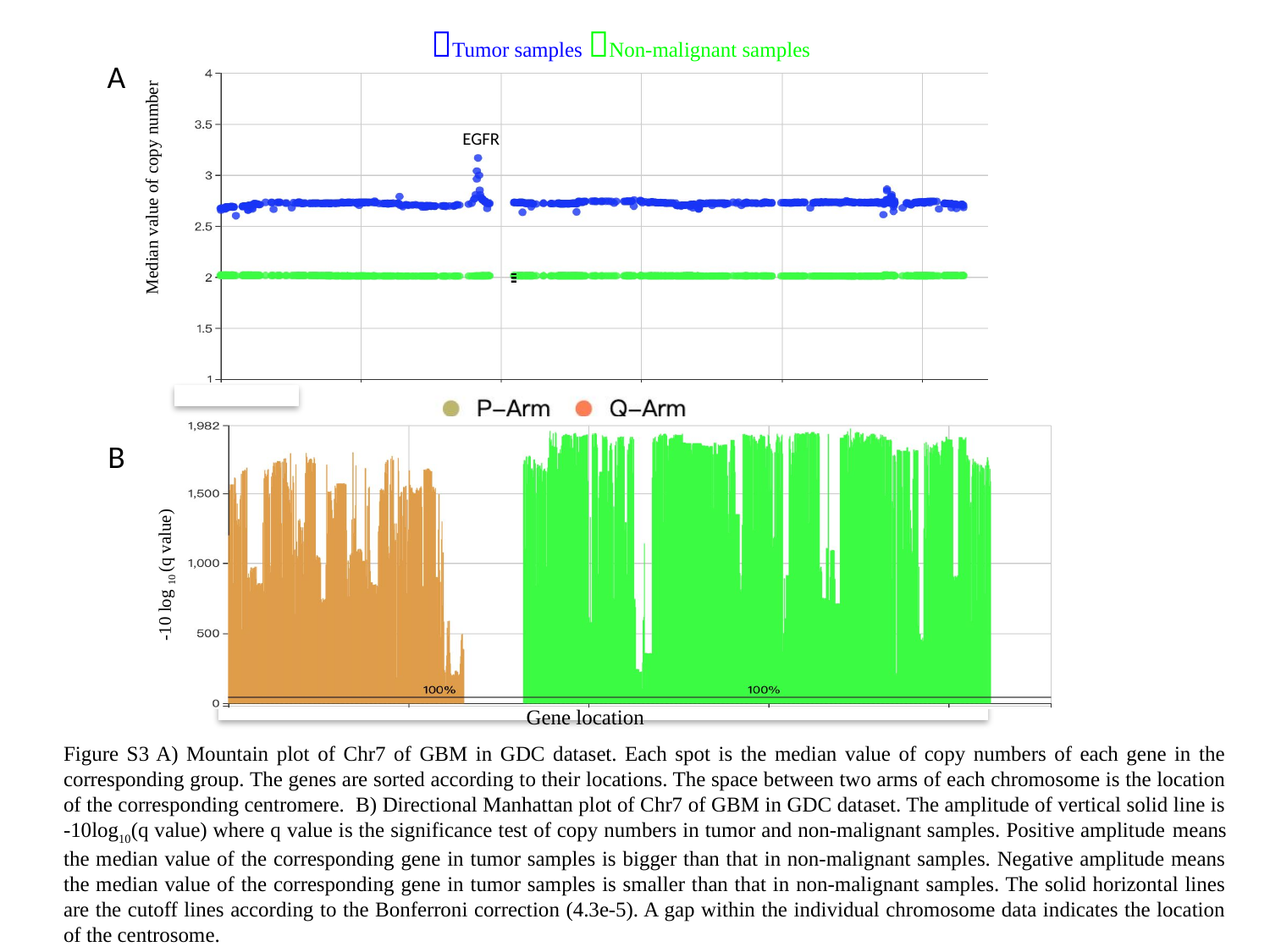

Tumor samples Non-malignant samples
A
Median value of copy number
EGFR
B
-10 log 10 (q value)
Gene location
Figure S3 A) Mountain plot of Chr7 of GBM in GDC dataset. Each spot is the median value of copy numbers of each gene in the corresponding group. The genes are sorted according to their locations. The space between two arms of each chromosome is the location of the corresponding centromere. B) Directional Manhattan plot of Chr7 of GBM in GDC dataset. The amplitude of vertical solid line is -10log10(q value) where q value is the significance test of copy numbers in tumor and non-malignant samples. Positive amplitude means the median value of the corresponding gene in tumor samples is bigger than that in non-malignant samples. Negative amplitude means the median value of the corresponding gene in tumor samples is smaller than that in non-malignant samples. The solid horizontal lines are the cutoff lines according to the Bonferroni correction (4.3e-5). A gap within the individual chromosome data indicates the location of the centrosome.

## Slide 4
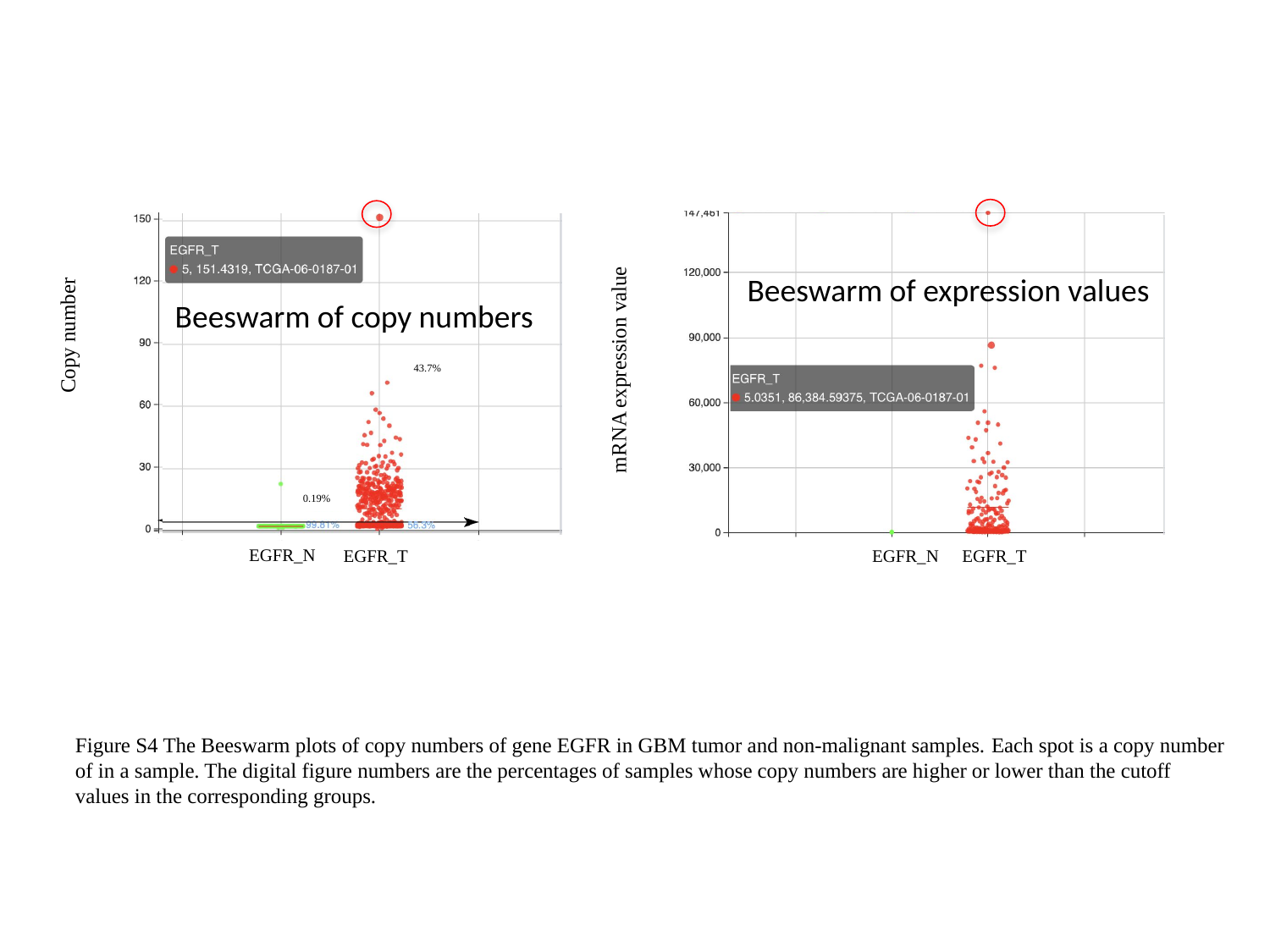

mRNA expression value
Beeswarm of expression values
Copy number
Beeswarm of copy numbers
43.7%
9.49%
0.19%
EGFR_N
EGFR_T
EGFR_N
EGFR_T
Figure S4 The Beeswarm plots of copy numbers of gene EGFR in GBM tumor and non-malignant samples. Each spot is a copy number of in a sample. The digital figure numbers are the percentages of samples whose copy numbers are higher or lower than the cutoff values in the corresponding groups.

## Slide 5
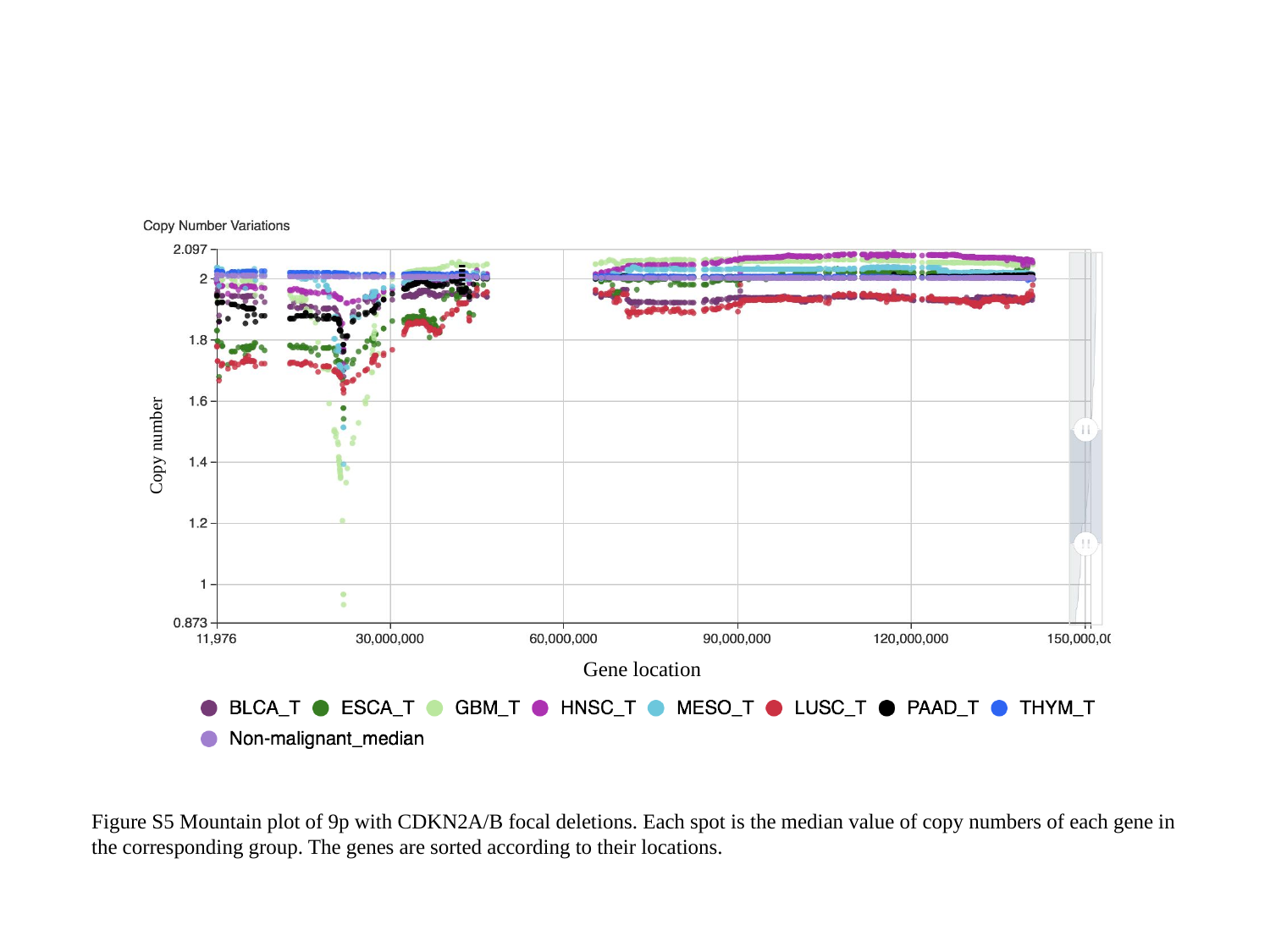

Copy number
Gene location
Figure S5 Mountain plot of 9p with CDKN2A/B focal deletions. Each spot is the median value of copy numbers of each gene in the corresponding group. The genes are sorted according to their locations.

## Slide 6
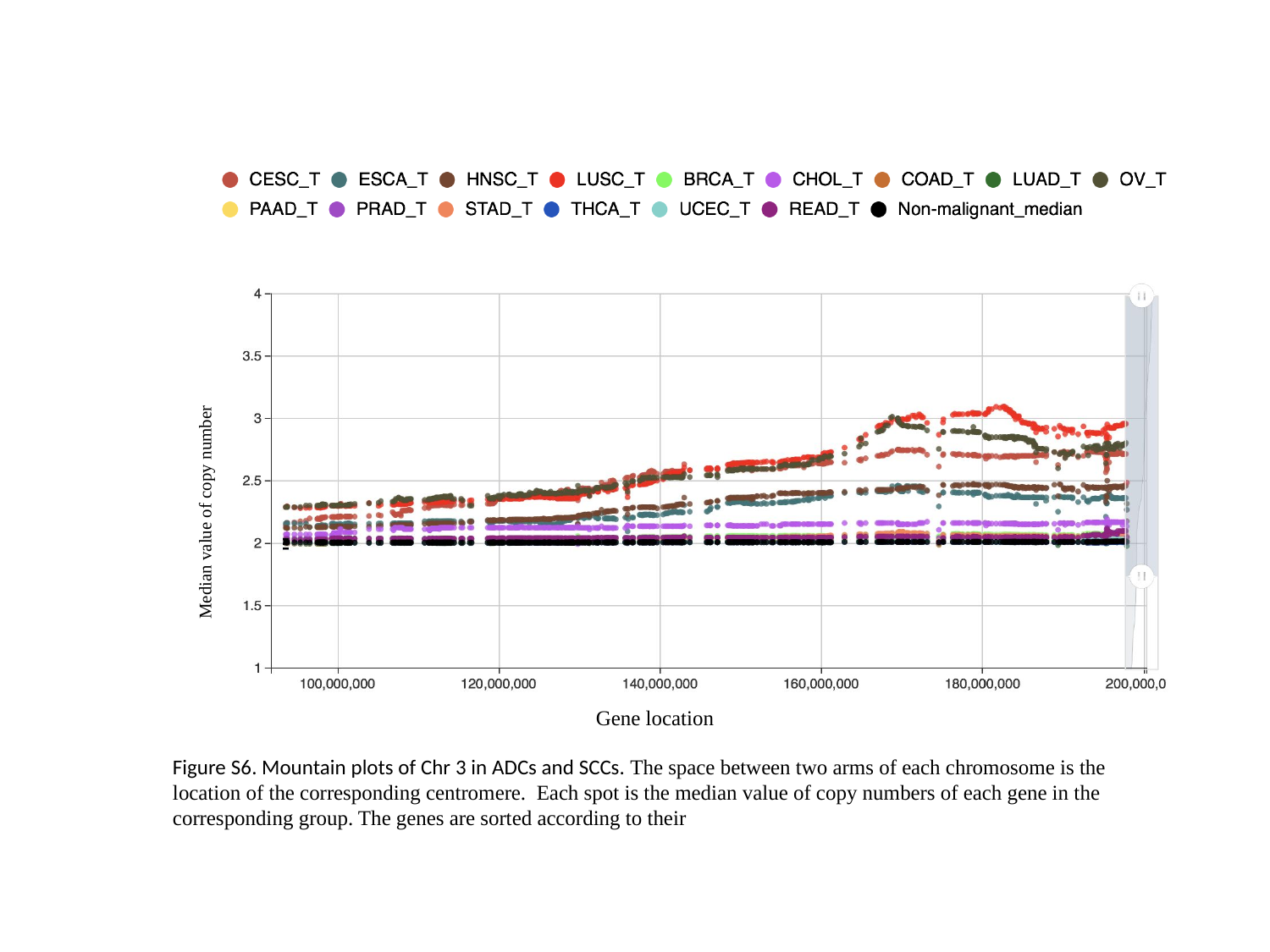

Median value of copy number
Gene location
Figure S6. Mountain plots of Chr 3 in ADCs and SCCs. The space between two arms of each chromosome is the location of the corresponding centromere. Each spot is the median value of copy numbers of each gene in the corresponding group. The genes are sorted according to their

## Slide 7
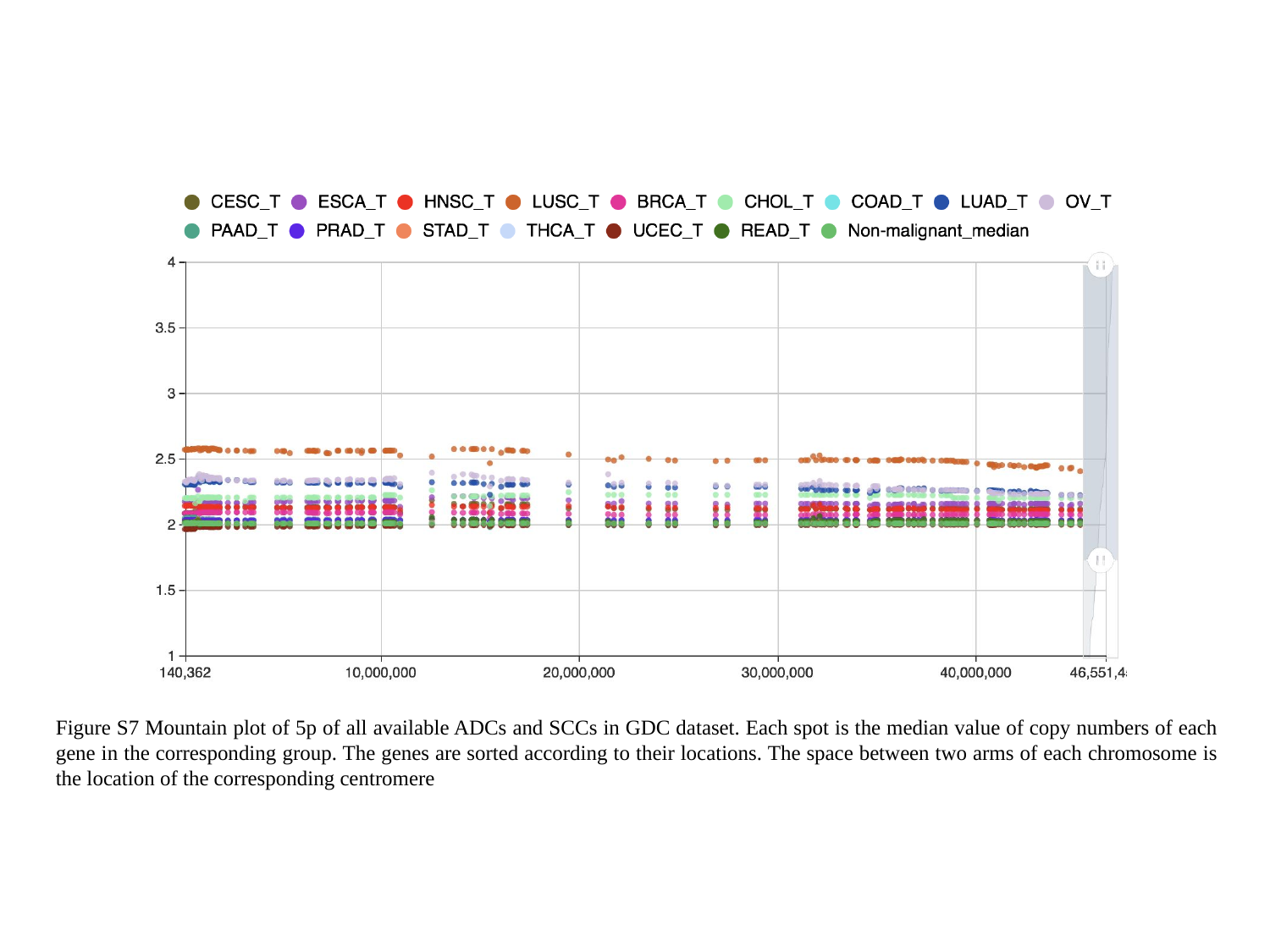

Figure S7 Mountain plot of 5p of all available ADCs and SCCs in GDC dataset. Each spot is the median value of copy numbers of each gene in the corresponding group. The genes are sorted according to their locations. The space between two arms of each chromosome is the location of the corresponding centromere

## Slide 8
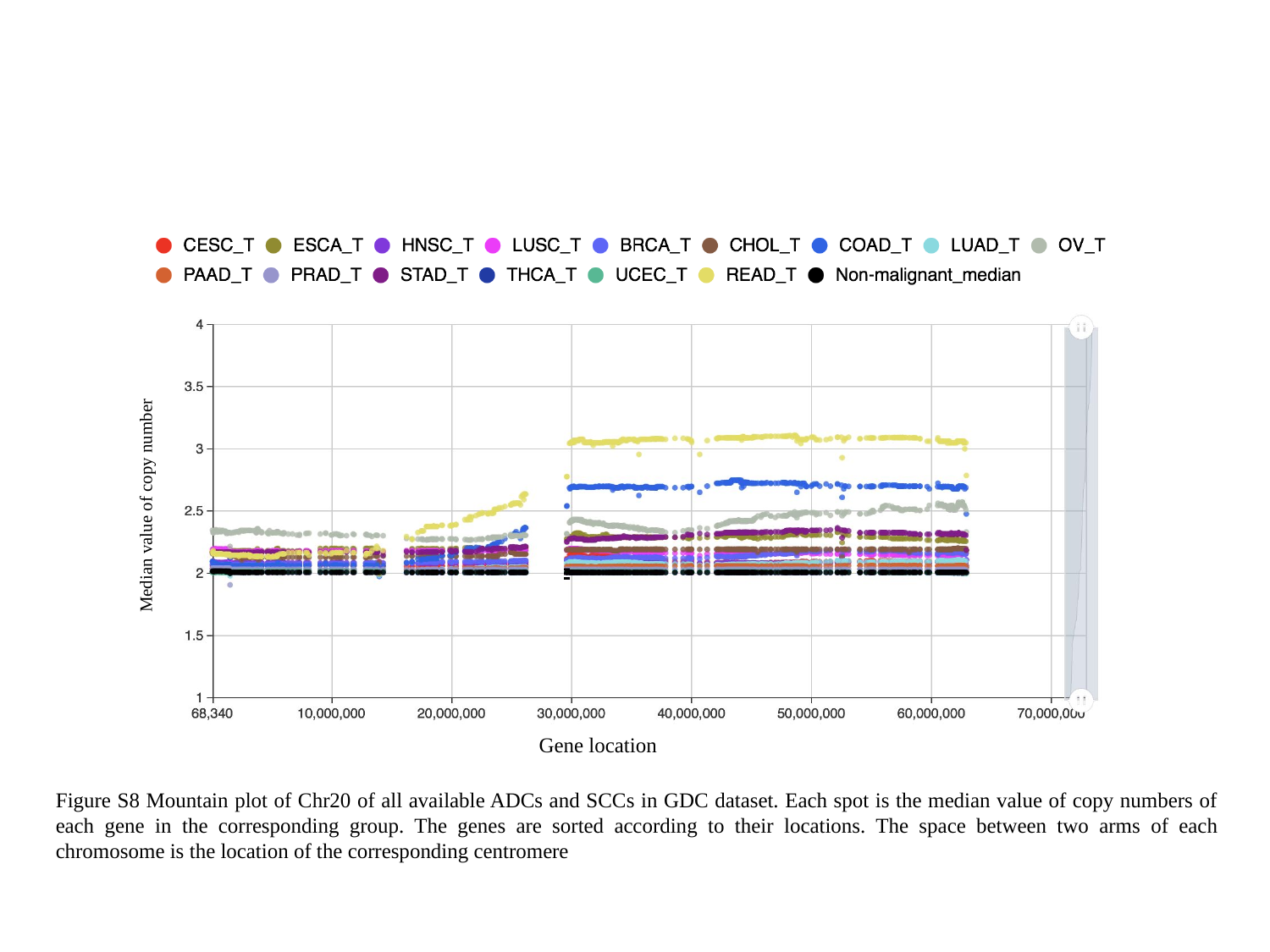

Median value of copy number
Gene location
Figure S8 Mountain plot of Chr20 of all available ADCs and SCCs in GDC dataset. Each spot is the median value of copy numbers of each gene in the corresponding group. The genes are sorted according to their locations. The space between two arms of each chromosome is the location of the corresponding centromere

## Slide 9
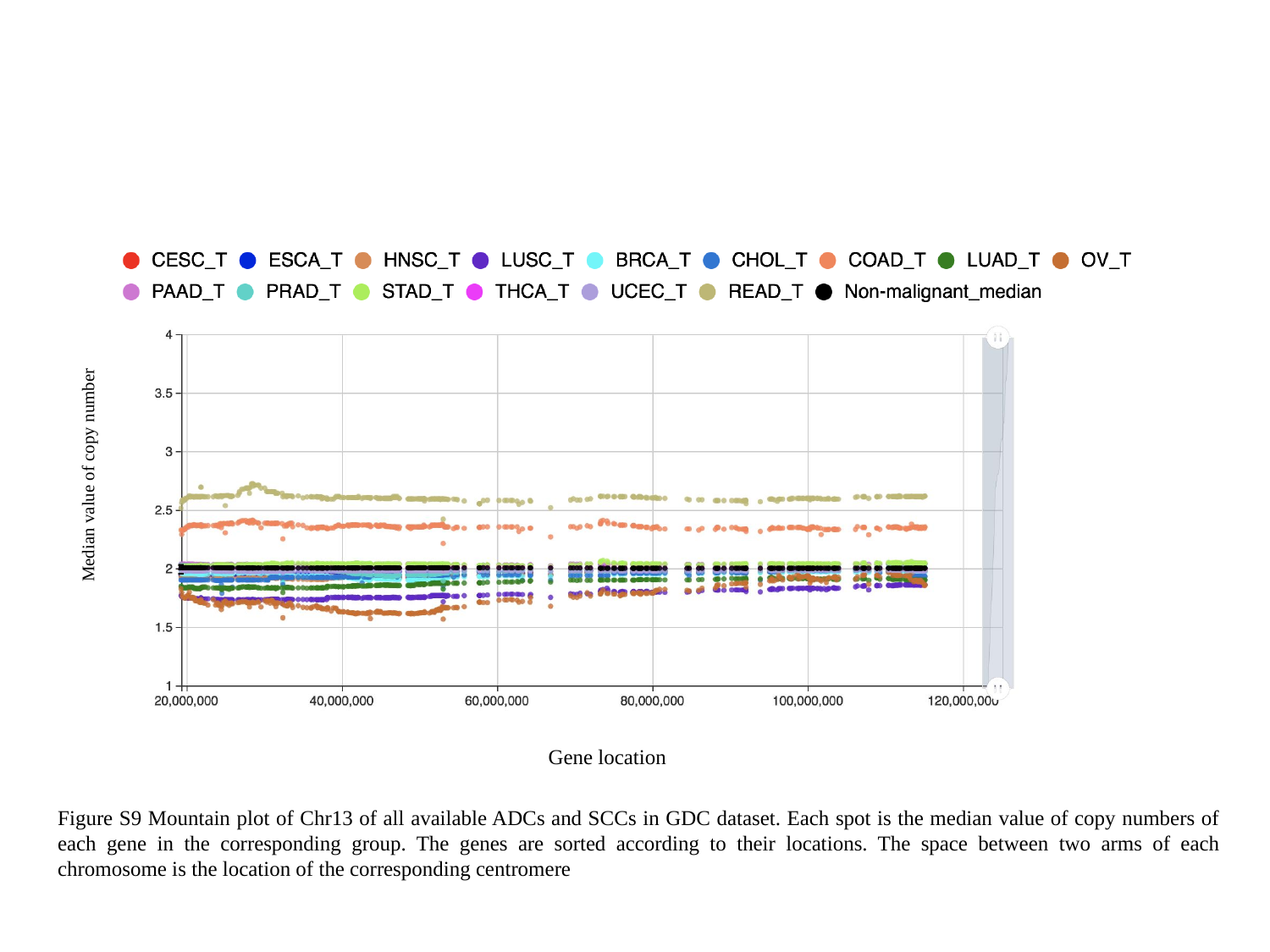

Median value of copy number
Gene location
Figure S9 Mountain plot of Chr13 of all available ADCs and SCCs in GDC dataset. Each spot is the median value of copy numbers of each gene in the corresponding group. The genes are sorted according to their locations. The space between two arms of each chromosome is the location of the corresponding centromere

## Slide 10
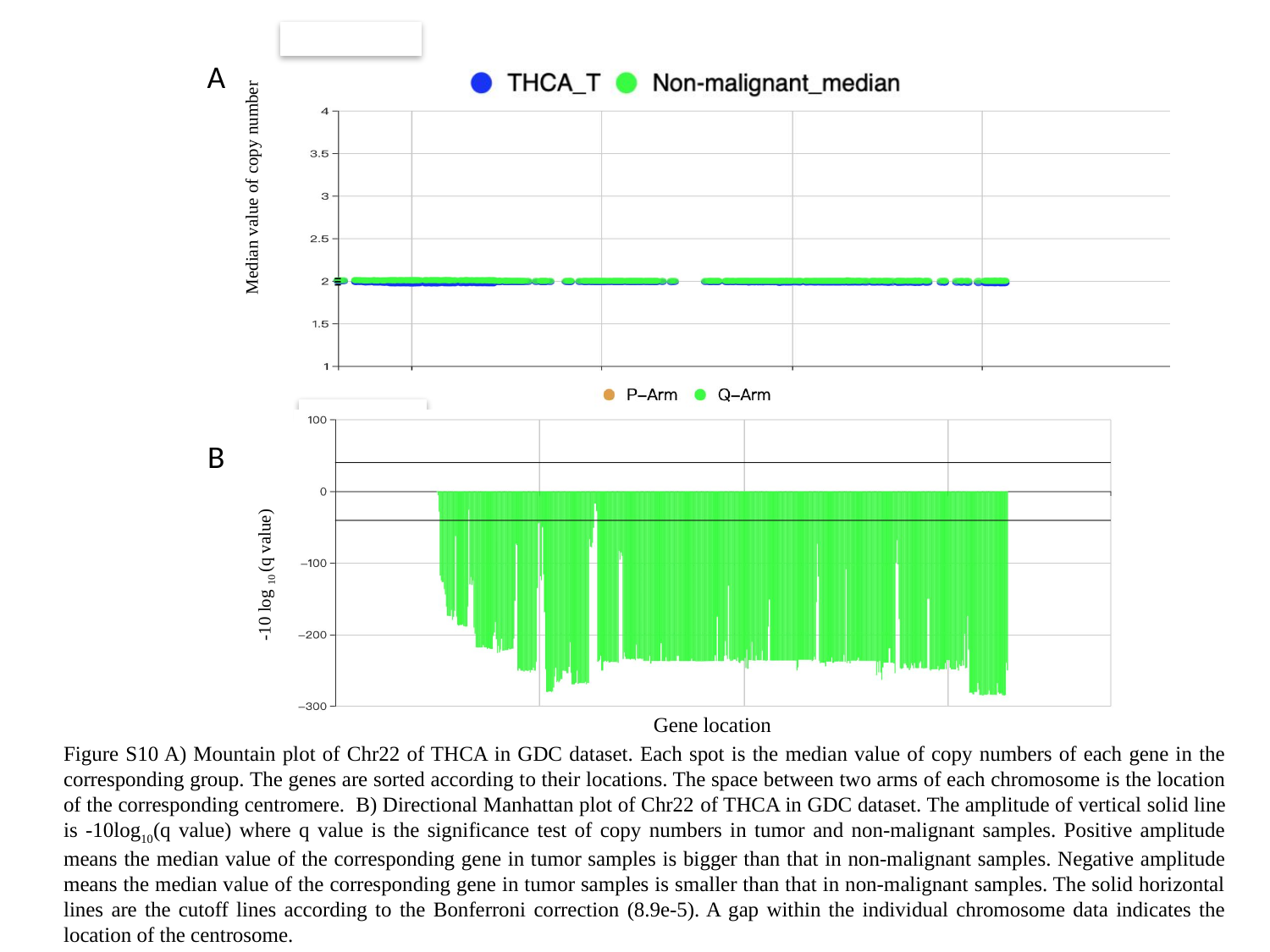

A
Median value of copy number
B
-10 log 10 (q value)
Gene location
Figure S10 A) Mountain plot of Chr22 of THCA in GDC dataset. Each spot is the median value of copy numbers of each gene in the corresponding group. The genes are sorted according to their locations. The space between two arms of each chromosome is the location of the corresponding centromere. B) Directional Manhattan plot of Chr22 of THCA in GDC dataset. The amplitude of vertical solid line is -10log10(q value) where q value is the significance test of copy numbers in tumor and non-malignant samples. Positive amplitude means the median value of the corresponding gene in tumor samples is bigger than that in non-malignant samples. Negative amplitude means the median value of the corresponding gene in tumor samples is smaller than that in non-malignant samples. The solid horizontal lines are the cutoff lines according to the Bonferroni correction (8.9e-5). A gap within the individual chromosome data indicates the location of the centrosome.

## Slide 11
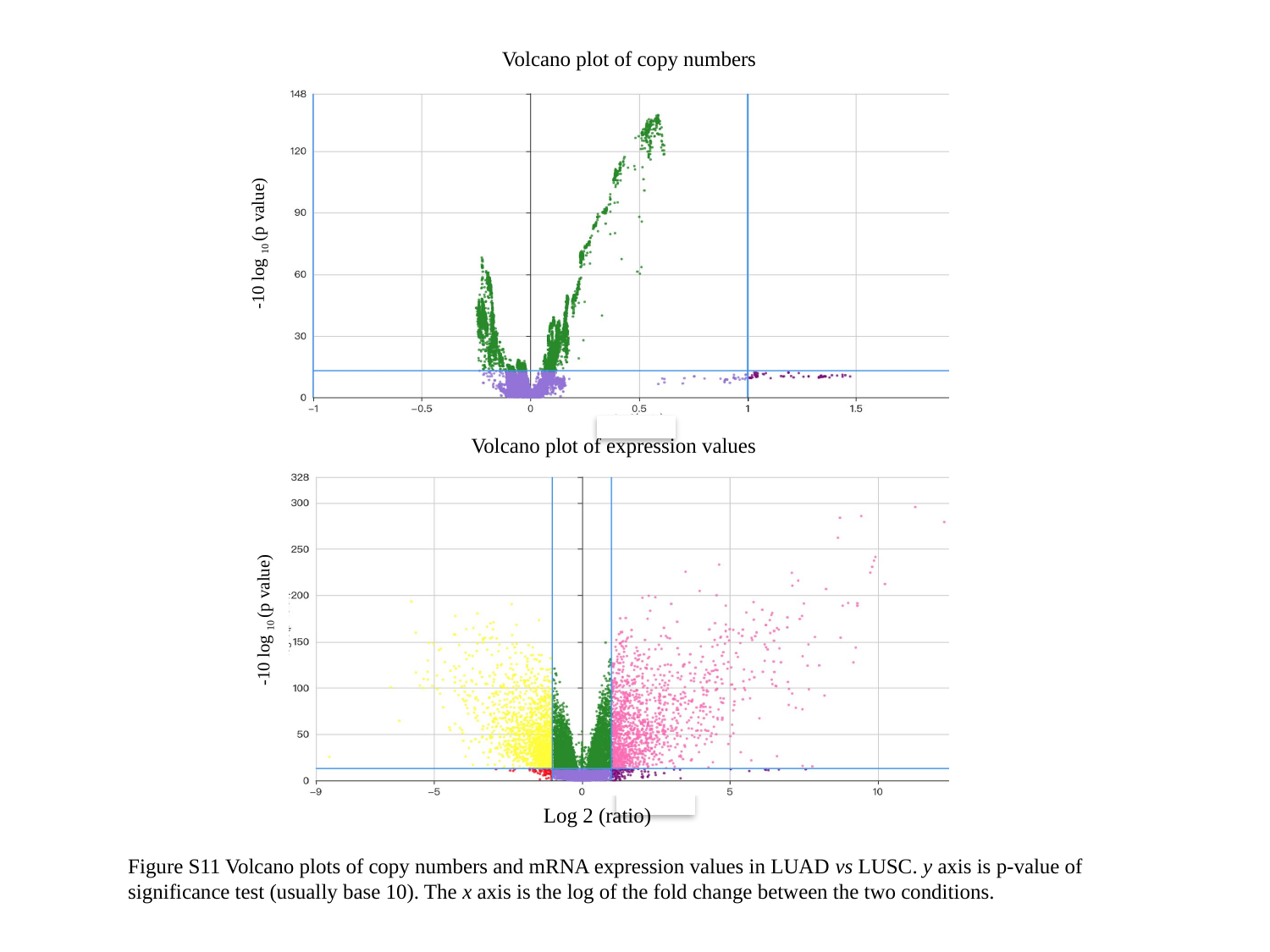

Volcano plot of copy numbers
-10 log 10 (p value)
Volcano plot of expression values
-10 log 10 (p value)
Log 2 (ratio)
Figure S11 Volcano plots of copy numbers and mRNA expression values in LUAD vs LUSC. y axis is p-value of significance test (usually base 10). The x axis is the log of the fold change between the two conditions.
